# Transcriptome analysis of heat resistance regulated by quorum sensing system in *Glaesserella parasuis*

**DOI:** 10.3389/fmicb.2022.968460

**Published:** 2022-08-11

**Authors:** Bingzhou Zhang, Changsheng Jiang, Hua Cao, Wei Zeng, Jingping Ren, Yaofang Hu, Wentao Li, Qigai He

**Affiliations:** ^1^State Key Laboratory of Agricultural Microbiology, College of Animal Sciences and Veterinary Medicine, Huazhong Agricultural University, Wuhan, China; ^2^The Cooperative Innovation Center for Sustainable Pig Production, Huazhong Agricultural University, Wuhan, China; ^3^Hubei Hongshan Laboratory, Wuhan, China

**Keywords:** quorum sensing, *Glaesserella parasuis*, transcriptome, heat shock resistance, molecular mechanism

## Abstract

The ability of bacteria to resist heat shock allows them to adapt to different environments. In addition, heat shock resistance is known for their virulence. Our previous study showed that the AI-2/luxS quorum sensing system affects the growth characteristics, biofilm formation, and virulence of *Glaesserella parasuis*. The resistance of quorum sensing system deficient *G. parasuis* to heat shock was obviously weaker than that of wild type strain. However, the regulatory mechanism of this phenotype remains unclear. To illustrate the regulatory mechanism by which the quorum sensing system provides resistance to heat shock, the transcriptomes of wild type (GPS2), ΔluxS, and *luxS* complemented (C-luxS) strains were analyzed. Four hundred forty-four differentially expressed genes were identified in quorum sensing system deficient *G. parasuis*, which participated in multiple regulatory pathways. Furthermore, we found that *G. parasuis* regulates the expression of *rseA*, *rpoE*, *rseB*, *degS*, *clpP*, and *htrA* genes to resist heat shock *via* the quorum sensing system. We further confirmed that *rseA* and *rpoE* genes exerted an opposite regulatory effect on heat shock resistance. In conclusion, the findings of this study provide a novel insight into how the quorum sensing system affects the transcriptome of *G. parasuis* and regulates its heat shock resistance property.

## Introduction

*Glaesserella parasuis* (*G. parasuis*) causes serious Glässer’s disease, which is characterized by severe infection of the upper respiratory tract, polyserositis, meningitis, and arthritis in pigs. It can lead to huge economic losses to the global pig industry (Liu et al., 2016). The quorum sensing system is a type of population density-dependent cell–cell signaling in bacteria that was first discovered and described in two luminous marine bacterial species, namely, *Vibrio fischeri* and *Vibrio harveyi* (Nealson and Hastings, 1979). Based on the differences in autoinducers, the quorum sensing system is classified into four types. The first type is luxR-I quorum sensing system, in which LuxI is responsible for the production of the N-acyl-homoserine-lactone (AHL) autoinducer; LuxR is activated by this autoinducer to increase the transcription of the luciferase operon. The second type is the autoinducer peptide (AIP, a kind of short peptide signaling) quorum sensing system that exists in gram-positive bacteria. The third type is the luxS/AI-2 quorum sensing system that is present in approximately half of all the sequenced bacterial genomes including gram-negative and gram-positive bacteria (Waters and Bassler, 2005). The fourth type is the AI-3/epinephrine/norepinephrine quorum sensing system (Kendall and Sperandio, 2007). The quorum sensing system has been implicated in the regulation of several physiological activities and abilities in bacteria such as virulence, antibiotic production, symbiosis, motility, and biofilm formation (Miller and Bassler, 2001). We have previously reported that the virulence and abilities of autoagglutination, adherence to PK-15 cells, and hemagglutination were significantly decreased in the *luxS* mutant strain of GPS2. Furthermore, the tolerance to heat shock stress was reduced in the *luxS* mutant strain. However, the ability of the *luxS* mutant strain to form biofilm was significantly increased (Zhang et al., 2019). While the regulatory mechanism of quorum sensing system in *G. parasuis* remains unclear. RNA-seq has emerged as a very important tool that is commonly used to study pathogen-host interactions, antibiotic resistance, and quorum sensing system (Wen et al., 2011; Liu et al., 2012; Fu et al., 2018). Therefore, RNA-seq was used to identify the potential regulatory mechanism of quorum sensing system in *G. parasuis*.

Several studies have reported the significance of the luxS/AI-2 quorum sensing system in antibiotic production, biofilm formation, virulence, and carbohydrate metabolism (Xue et al., 2013; Ma et al., 2017; Abisado et al., 2018). However, the regulation of heat shock resistance by the luxS/AI-2 quorum sensing system is rarely reported. The ability of pathogenic microorganisms to heat shock resistance is crucial for adapting to different environments. In addition, it affects the virulence of pathogenic microorganisms. The *LuxS* gene decreases the resistance of *Porphyromonas gingivalis* to heat stress (Yuan et al., 2005). Although we have previously reported that the *luxS* gene could significantly increase the tolerance of heat shock stress in *G. parasuis* (Zhang et al., 2019). While the regulatory mechanism of heat shock resistance is still unclear in *G. parasuis*. HtrA is a heat shock-induced serine protease with homologs present in a wide range of bacteria and eukaryotes (Pallen and Wren, 1997). The *HtrA* gene is known to affect the bacterium’s ability to withstand heat and other stress and virulence in *Bacillus anthracis* and *Salmonella typhimurium* (Pallen and Wren, 1997; Mutunga et al., 2004; Chitlaru et al., 2011). In *G. parasuis*, the *htrA* gene has been reported to increase the ability to heat shock resistance (Zhang et al., 2016). In *Escherichia coli*, RseB is a periplasmic protein that negatively regulates σ^E^ (RpoE) activity and specifically interacts with RseA, an inner membrane protein. In contrast, RseC is an inner membrane protein that positively modulates the activity of σ^E^, which is a heat shock transcription factor (Missiakas et al., 1997). DegS is a periplasmic protease anchored to the inner-membrane, whereas ClpP is a cytoplasmic protease; both work together to cleave RseA and release σ^E^ from the RseA-σ^E^ compound to the cytoplasm, thus initiating the transcription of heat shock stress-related genes to resist heat shock stress (Kim, 2015). Therefore, we wanted to study whether this regulatory mechanism of heat shock resistance also exists in *G. parasuis*.

To the best of our knowledge, no study has been conducted to study the regulatory mechanism of heat shock resistance by the quorum sensing system in *G. parasuis*. Therefore, this study aims to illustrate the regulatory network of the quorum sensing system in *G. parasuis* and identify the potential mechanism of heat resistance in *G. parasuis* by RNA-seq and analyze the differentially expressed genes in the transcriptome.

## Materials and methods

### Bacterial strains, plasmids, primers, and culture conditions

The bacterial strains and plasmids used in this study are listed in Table 1. GPS2 is the standard reference strain of *G. parasuis* serotype 2, ΔluxS and C-luxS are *luxS* gene mutant strain and complemented strain, respectively, which were constructed and preserved in our laboratory (Zhang et al., 2019). ΔrseA and ΔrpoE are *rseA* and *rpoE* gene mutant strains of GPS2, respectively. All bacterial strains were grown in Tryptic Soy Broth (TSB) medium or Tryptic Soy Agar (TSA) plate (Difco Laboratories, Detroit, MI, United States) supplemented with 10 μg/ml nicotinamide adenine dinucleotide (NAD) and 5% (v/v) inactivated cattle serum (T/V/S) (Zhejiang Tianhang Biotechnology, Zhejiang, China) at 37°C. Kanamycin (50 μg/ml) and gentamicin (20 μg/ml) (Sigma-Aldrich, Missouri, United States) was added to the medium of mutant and complemented strains, respectively. The plasmid pK18mobsacB was used to construct the GPS2 mutant strain using the natural transformation method. The primers used in the construction of GPS2 mutant strain and qPCR are listed in Table 2.

**Table 1:** Characteristics of bacterial strains and plasmids used in this study.

Strain/plasmid	Characteristics and/or sequences	Source/reference
**Strain**		
GPS2	*Glaesserella parasuis* reference strain of serotype 2	Preserved in our laboratory
ΔluxS	*luxS* gene mutant strain of GPS2, Kan^r^	Zhang et al. (2019)
C-luxS	The complemented strain of ΔluxS of GPS2, Kan^r^, Gm^r^	Zhang et al. (2019)
ΔrseA	*resA* gene mutant strain of GPS2, Kan^r^	This study
ΔrpoE	*rpoE* gene mutant strain of GPS2, Kan^r^	This study
**Plasmids**		
pK18mobsacB	Suicide and narrow broad-host vector, Kan^r^	Preserved in our laboratory
pK18-ΔrseA	A 1986 bp overlap fragment containing Kan^r^, the upstream and downstream sequences of the *resA* gene in pK18mobsacB, Kan^r^	This study
pK18-ΔrpoE	A 2046 bp overlap fragment containing Kan^r^, the upstream and downstream sequences of the *resA* gene in pK18mobsacB, Kan^r^	This study

**Table 2:** The primers used in this study.

Primer	Sequence (5′–3′)	Tm (°C)	Size (bp)
**Primers used for construction of ΔrseA and ΔrpoE mutant strains:**			
RseA-uF	CCG**GAATTC**ACCGCTTGTGGTGATAAGAAAGCGTTTAA	66.5	549
RseA-uR	TTATCTTGTGCAATGAGATAACTCCAAAGTATTTTAATTC	58.7	
RseA-KF	GAATTAAAATACTTTGGAGTTATCTCATTGCACAAGATAA	58.7	909
RseA-KR	CGCCCCCAGAGTGATAAGAAGCAATTAACCAATTCTGATTAG	65.4	
RseA-dF	CTAATCAGAATTGGTTAATTGCTTCTTATCACTCTGGGGGCG	65.4	528
RseA-dR	TGC**TCTAGA**ACAAGCGGTACTTCACAAATGGCTTCTTCC	66.9	
RpoE-uF	CCG**GAATTC**ACCGCTTGTTATCGCAAGGGACATTTATC	66.2	679
RpoE-uR	TTATCTTGTGCAATGCGTTTTGCGTTCTCCTAACTTCTAC	65.0	
RpoE-KF	GTAGAAGTTAGGAGAACGCAAAACGCATTGCACAAGATAA	65.0	909
RpoE-KR	ATAACTCCAAAGTATTTTAATTCTGCAATTAACCAATTCTGATTAG	60.0	
RpoE-dF	CTAATCAGAATTGGTTAATTGCAGAATTAAAATACTTTGGAGTTAT	60.0	558
RpoE-dR	TGC**TCTAGA**ACAAGCGGTTGCACTAGTACGACGTTGT	68.4	
**Primers used for verification of ΔrseA and ΔrpoE mutant strains:**			
RseA-F	ATGCAACATAAAGAAACACTTTCCG	54.7	630
RseA-R	TTACTGATTATTTTGCGGTTGTGCT	54.7	
RpoE-F	ATGAGTGAGCAAAGAGCCGATCAAG	59.6	570
RpoE-R	CTACAAAAGCGGATTGATTTTGGCA	56.3	
**Primers used for RT-qPCR:**			
pncB-F/R	ACGATCAACGAATGGCAGGT	57.0	157
	AAAATCGCATCGCTCGCTTC		
HAPS0219-F/R	ACCTTACGCCATTGCCTTGA	56.9	136
	CACACTATCCCCTGCGGTTT		
rcnB-F/R	GAGATGATTGAGCCGACGGT	56.7	111
	TTGTGCGCCTAAAGACAAGC		
rcnA-F/R	AGGCAGGGCTGAGCTTAATG	57.1	186
	TCGTCACTAACCCAATCGCC		
rseA-F/R	AGCGTGATGCGAAGTGAAGA	57.1	154
	GTGTTGCCCAGCGTTTCAAT		
HAPS2254-F/R	ATTAGCAGACGGTGAGCTGG	56.9	154
	TAAAGCGGTGTCGTTTCCGA		
HAPS1551-F/R	AACGCTATTCACCGCCAGTT	57.1	105
	AGTCGAGTACGGCGTAGGTA		
aspA-F/R	TGCGGAAGTCACCCAGTTAC	57.0	101
	TACGTTTTAACGCCCCGTGA		
htrA-F/R	CGATGCAGCAGTAAACCGTG	57.1	115
	CGCAATACCTGCATTACCGC		
acnB-F/R	TCGAATGCTATTGCCCGACA	57.0	103
	CGCAAAGGCAACTAAGCCTG		
HAPS2130-F/R	CAGCTTGATCGCCTGATTGC	57.1	141
	CGGTAACATCATTGCTGCGG		
icd-F/R	TGGACTGAAGCGGCAGATTT	57.0	109
	TTGTACGGAGTGTTGCTCCC		
ulaA-F/R	GGTATGGCTGACTGGGCATT	57.1	141
	TTCTGCTTCTTCAGCGCGTA		
gltA-F/R	ACCCAATGGCGGTTATGTGT	56.9	136
	ATAGCTGCAAGGGTTGGCAT		
HAPS1218-F/R	AACAACGATTGGCGGTGTTG	57.1	102
	TCCCATTCAGGATCGTTGCC		
mlc-F/R	CCGTTCCACTGGCGTTATCT	57.1	187
	CGTTGGTAAGCGGTGTGTTG		
rpoE-F/R	TGGCATCAATCGCTTCCCTT	60.2	122
	AACGGCAATTACGTTGCGTG		
rseB-F/R	ATGGCAGCTAAAATGGGTGC	59.4	197
	CACTGGTTTTTCCCTCACGC		
DegS-F/R	TAGCCTCGAATAACACGCCC	60.0	177
	GGGTAATTCAGGCGGAGCTT		
clpP-F/R	TAATTCGCCAGGTGGTGTCG	60.2	146
	AACGCTTACCTTTTGCCCCT		
16S rRNA-F/R	AAGAAGCACCGGCTAACTCC	57.0	121
	CGGGGCTTTCACATCTCACT		

Bases labeled in bold are restriction enzymes, underline labeled bases are sequences of USS.

### Sample preparation for RNA-seq

Strains GPS2, ΔluxS and C-luxS were cultured in the T/V/S medium at 37°C with 180 rpm. When the cultures had reached the end of the logarithmic phase, the total RNA of each sample was extracted using the Trizol reagent (Invitrogen, Carlsbad, CA, United States), according to the manufacturer’s instructions. Afterward, recombinant DNase I and RNase inhibitor (Takara, Dalian, China) was used to remove the DNA from extracted RNA samples based on the manufacturer’s instructions, following which the concentration and quality of the extracted RNA were measured. Afterward, the qualified samples were sent to Novogene Bioinformatics Technology Co., Ltd. (Beijing, PR China) for further RNA quantification, library preparation, clustering, sequencing, and transcriptomic analysis. The sequencing platform was Illumina HiSeq 2000 and the Illumina PE150 sequencing strategy was used in this study.

### Transcriptomic analysis

Preliminary quality control including removal of adaptors and poor-quality reads was performed. Next, Bowtie 2 2.2.3 was used in clean data mapping based on the SH0165 genome sequence and annotation files published in the NCBI genome database.^1^ Mapped reads were filtered based on mapping quality, and only uniquely mapped reads (mapping quality >10) were used for further analysis. To quantify the expression level of genes, the read numbers mapped to each gene were counted using the HTSeq v0.6.1. A principal component analysis (PCA) plot was used to assess the similarity and suitability of biological replicates before performing differential expression analysis using DESeq 1.10.1. A transcript was considered to have significant DE if the false discovery rate (FDR) was ≤0.05. The expression of one gene was considered as up-regulated if log_2_ fold change ≥1 and FDR < 0.05. In turn, the expression was considered down-regulated (log_2_ fold change < −1 and FDR < 0.05). In the end, Gene Ontology (GO)^2^ was used for the functional classification of differentially expressed genes. Kyoto Encyclopedia of Genes and Genomes (KEGG) Pathway^3^ was utilized to identify the participated pathways of differentially expressed genes.

### RT-qPCR verification and analyses

The RNA samples used for RT-qPCR were extracted as described above. For each sample, cDNA was synthesized using the HiScript Q RT SuperMix (Vazyme, China). Real-time PCR was performed using the ViiA™ 7 Real-Time PCR system and Hieff™ qPCR SYBR® Green Master Mix (Low Rox Plus) (YEASEN, Shanghai, CHN). 16S rRNA was amplified as endogenous control and the results were analyzed using the 2^−ΔΔCt^ method in triplicates in three independent experiments. The primers used in RT-qPCR are listed in Table 2.

### Construction and verification of ΔrseA and ΔrpoE mutant strains

All plasmids, strains, and primers used for the construction of ΔrseA/ΔrpoE are listed in Tables 1, 2. The construction and verification of ΔrseA and ΔrpoE mutant strains were performed as described in previous studies with certain modifications (Zhang et al., 2019). Briefly, the upstream (549 bp/679 bp) and downstream (528 bp/558 bp) fragments of *rseA/rpoE* gene from the GPS2 genome and kanamycin resistance cassette (909 bp) from pSHK3 plasmid were amplified using primer pairs RseA-uF (uR)/RpoE-uF (uR), RseA-dF (dR)/RpoE-dF (dR), and RseA-KF (KR)/RpoE-KF (KR), respectively. Next, the overlap extension method was used to obtain a new fragment (UKD, *rseA*/*rpoE* gene upstream sequence, kanamycin resistance cassette sequence, and *rseA*/*rpoE* gene downstream sequence) and the obtained fragment was inserted into the pk18mobsacB plasmid with *EcoR*I and *Xba*I restriction enzymes to generate the recombinant plasmid pk18-rseA/rpoE-UKD. Afterward, the recombinant plasmid was introduced into GPS2 by natural transformation method as described in previous studies with simple modifications (Zhang et al., 2012; Wang et al., 2013; Zou et al., 2013). Briefly, 20 μl of cAMP (8 mM) was added to 20 μl of recipient bacterial suspension in the logarithmic phase (OD_600 nm_ = 0.9). Ten minutes later, 2 μg of the donor DNA plasmid was added to the bacterial mixture. Subsequently, the cells were transferred to the T/V/S solid medium and incubated at 37°C for 6 h. At last, cells were transferred to a kanamycin (50 μg/ml) selective plate and the cells were incubated at 37°C for 48 h for further identification. To confirm the construction results of ΔrseA/ΔrpoE, the UKD sequence of *rseA* and *rpoE*, kanamycin resistance cassette sequence, and *rseA* or *rpoE* gene were amplified and verified by sequencing.

### Heat shock assay

The heat shock assay was performed using a previously described method (Xie et al., 2013; Huang et al., 2016; Zhang et al., 2019) with some modifications. The OD_600 nm_ value of overnight cultured GPS2, ΔluxS, C-luxS, ΔrseA, and ΔrpoE strains was adjusted to 0.8. Afterward, cells were incubated in a 48°C water bath for 30 min. Meanwhile, untreated cell suspensions of each strain were incubated at 37°C for 30 min were used as a control. Following incubation, the cultures were serially diluted by sterile phosphate-buffered saline (PBS), and their colony-forming units (CFUs) were determined by plate counting. The survival rate of heat-treated cells was calculated as the concentration of bacteria in the heat shock group (CFU/mL) / the concentration of bacteria in control group (CFU/mL) × 100%. The assay was independently performed thrice in triplicate.

### Statistical analysis

The results are presented as means ± standard deviation (SD). The statistical analysis was performed using the two-tailed Student’s *t-*test in GraphPad Prism 7.0 (GraphPad Software Inc., United States). The significant difference was defined as *p* < 0.05 (*), *p* < 0.01 (**), *p* < 0.001 (***), respectively. The “ns” means no significant difference.

## Results

### Transcriptome sequencing and annotation

To provide evidence that the quorum sensing system could affect the heat resistance of *G. parasuis* at the level of gene transcription, RNA-seq was conducted to compare the transcriptional profiling of GPS2, ΔluxS, and C-luxS strains. The results showed that 12,159,557 ± 862,999 raw reads and 12,037,669 ± 843,239 clean reads with a Q20 value of 98.15% ± 0.16% in the GPS2 group; 13,084,545 ± 203,701 raw reads and 12,172,199 ± 173,480 clean reads with a Q20 value of 98.28% ± 0.10% in the ΔluxS group; and 13,574,164 ± 421,580 raw reads and 13,061,193 ± 386,024 clean reads with a Q20 value of 98.25% ± 0.04% in the C-luxS group. In addition, the mapping ratios were 87.81% ± 0.28, 88.35% ± 0.09, 87.01% ± 0.13% in GPS2, ΔluxS, and C-luxS groups, respectively. The Pearson correlation coefficient in GPS2, ΔluxS, and C-luxS groups were all over 0.99. Therefore, the quality of sequencing results was very high and the data could represent the real differentially expressed genes very well.

### Differentially expressed genes among GPS2, ΔluxS, and C-luxS

**Figure 1:**
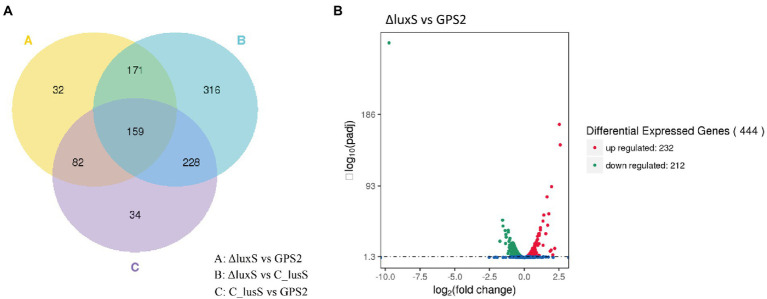
**(A)** Venn diagram of differentially expressed genes. “A” means differentially expressed genes between GPS2 and ΔluxS groups; “B” means differentially expressed genes between ΔluxS and C-luxS groups; “C” means differentially expressed genes between GPS2 and C-luxS groups. **(B)** Volcano plot of differentially expressed genes between ΔluxS and GPS2 groups. Red dots represent up-regulated genes; green dots represent down-regulated genes; and blue dots represent non-differentially expressed genes.

To more intuitively display the transcriptome data, differentially expressed genes among GPS2, ΔluxS, and C-luxS groups are presented in Figure 1A using the Venn diagram. The differentially expressed genes between ΔluxS and C-luxS, and between GPS2 and C-luxS groups were 874 and 503, respectively (Figure 1A). In addition, the Volcano plot of differentially expressed genes showed that the differentially expressed genes between GPS2 and ΔluxS groups were 444, among which 232 genes were up-regulated and 212 genes were down-regulated (Figure 1B). The transcriptome results of differentially expressed genes between GPS2 and ΔluxS groups are listed in Supplementary Table 1.

### Go annotation and KEGG pathway mapping of differentially expressed genes

**Figure 2:**
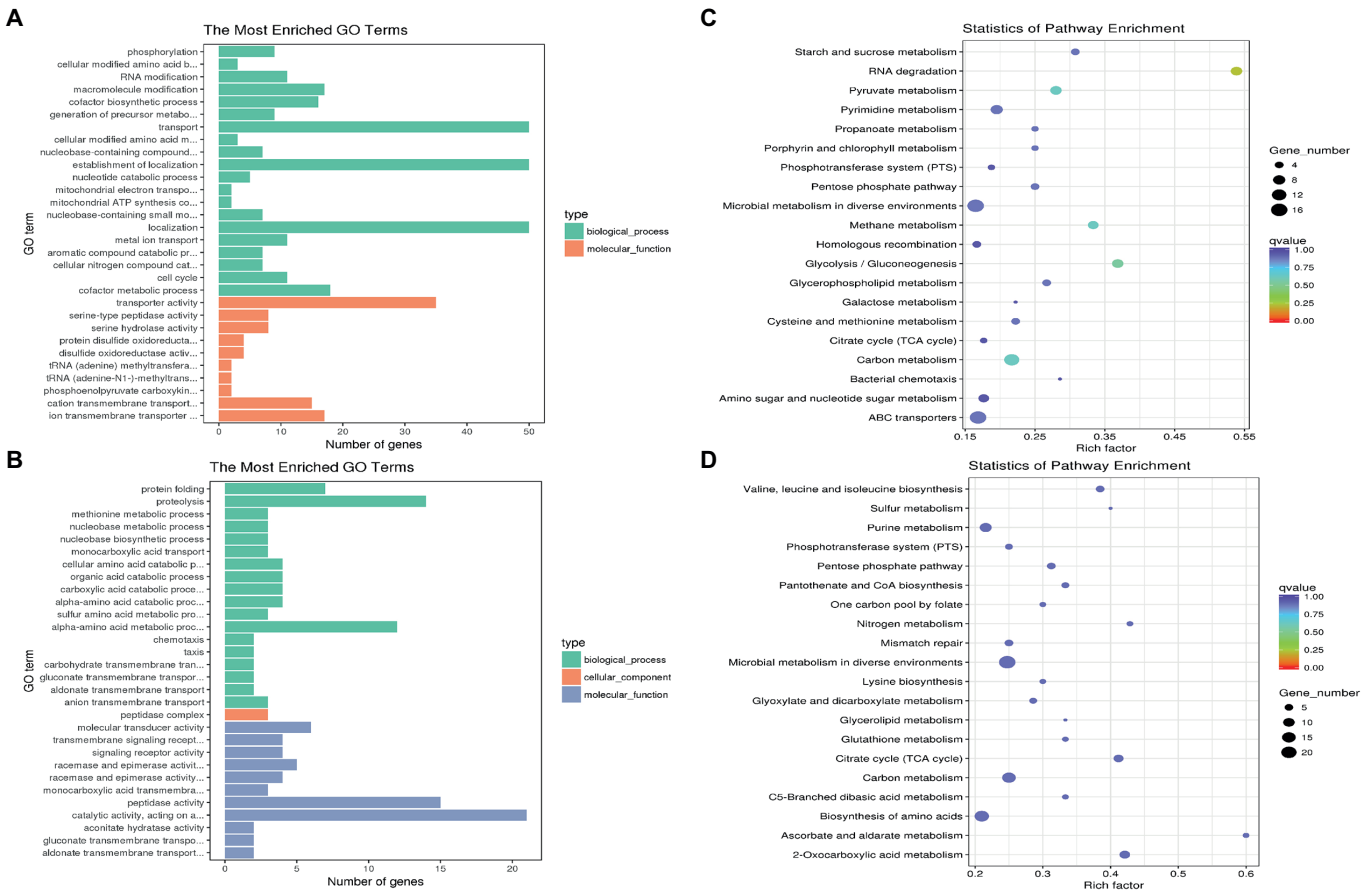
GO and KEGG pathway enrichment analysis of differentially expressed genes in ΔluxS and GPS2 groups. The numbers of genes that are up- **(A)** and down-regulated **(B)** in ΔluxS versus GPS2 were categorized according to role categories with GO enrichment analysis. KEGG pathway enrichment analysis of ΔluxS versus GPS2 up-regulated **(C)** and down-regulated **(D)** differentially expressed genes. The size of each point shows the number of genes in the pathway.

To illustrate the regulatory effect of the quorum sensing system on *G. parasuis*, 444 differentially expressed genes between GPS2 and ΔluxS groups were subjected to GO classification and KEGG pathway analysis to explore the potential functions of these differentially expressed genes. GO categories results showed that the up-regulated genes in the ΔluxS group primarily focused on biological process and molecular function, including transport, the establishment of localization, and localization in biological process, and transporter activity, ion transmembrane transporter, and cation transmembrane transporter in molecular function, compared with GPS2 (Figure 2A). In addition, the down-regulated genes in the ΔluxS group focused on biological process and molecular function, including proteolysis, alpha-amino acid metabolic process and protein folding in biological process, and catalytic activity, peptidase activity, and molecular transducer activity in molecular function, compared with GPS2 (Figure 2B).

According to the KEGG analysis, 444 differentially expressed genes between GPS2 and ΔluxS groups were annotated to be involved in 65 different pathways. Compared with GPS2, the most abundant pathways of up-regulated genes in the ΔluxS group were microbial metabolism in diverse environments, ABC transporters, and pyruvate metabolism (Figure 2C). The most abundant pathways of down-regulated genes in the ΔluxS group were microbial metabolism in diverse environments, biosynthesis of amino acids, and carbon metabolism (Figure 2D).

### RT-qPCR verification of differentially expressed genes

**Figure 3:**
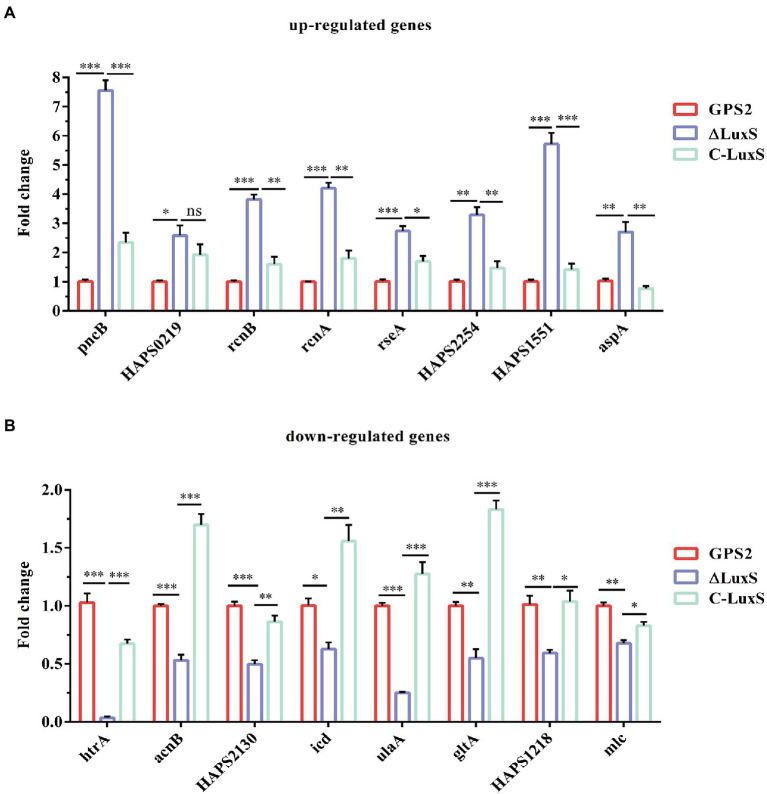
RT-qPCR verification. Eight up-regulated genes **(A)** and eight down-regulated genes **(B)** in RNA-seq results were selected for verification. The experimental data were calculated using the 2^−ΔΔCt^ method. All above assays were performed thrice in triplicate. Bars represent the mean ± standard deviation of three independent experiments. *p < 0.05, **p < 0.01, and ***p <  0.001.

To verify the sequencing results of differentially expressed genes, eight up-regulated genes and eight down-regulated genes in ΔluxS were selected randomly for RT-qPCR. The results of RT-qPCR confirmed that *pncB*, *HAPS_0219*, *rcnB*, *rcnA*, *rseA*, *HAPS_2254*, *HAPS_1551*, and *aspA* genes were up-regulated, and *htrA*, *acnB*, *HAPS_2130*, *icd*, *ulaA*, *gltA*, *HAPS_1218*, and *mlc* genes were down-regulated in the *luxS* mutant strain compared with the wild type strain, which were the same as the sequencing results. However, a little difference in fold changes of these genes between transcriptome and RT-qPCR analysis was observed (Figure 3).

### *rseA* and *rseB* genes exerted an opposite effect in regulating the toleration of heat shock resistance

**Figure 4:**
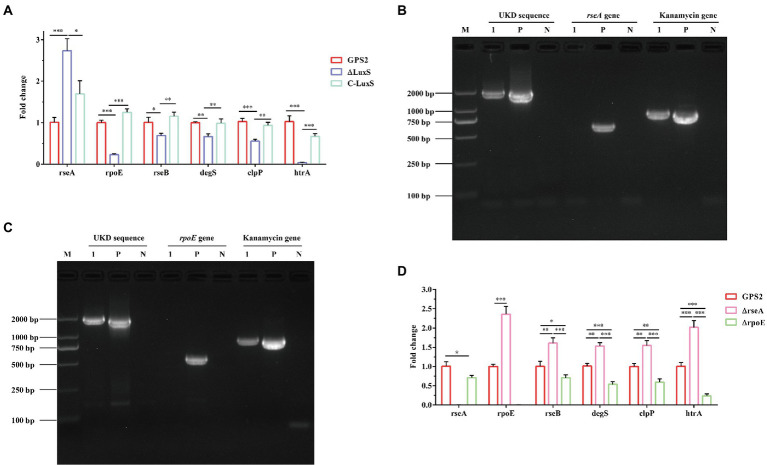
RT-qPCR verification of heat shock-related genes in *Glaesserella parasuis*. **(A)** The differential expression of *rseA*, *rpoE*, *rseB*, *degS*, *clpP*, and *htrA* genes in GPS2, ΔluxS, and C-luxS strains. All above assays were performed thrice in triplicate. Bars represent the mean ± standard deviation of three independent experiments. *p < 0.05, **p < 0.01, and ***p < 0.001. **(B)** PCR identification of the *rseA* mutant strain by amplification of the *rseA*-UKD fragment, kanamycin resistance cassette sequence fragment, and *rseA* gene fragment. **(C)** PCR identification of the *rpoE* mutant strain by amplification of the *rpoE*-UKD fragment, kanamycin resistance cassette sequence fragment, and the *rpoE* gene fragment. M: DL 2000 Mark, 1: *rseA* or *rpoE* mutant strain, P: positive control, and N: negative control. **(D)** The differential expression of *rseA*, *rpoE*, *rseB*, *degS*, *clpP*, and *htrA* genes in GPS2, ΔrseA, and ΔrpoE strains. All above assays were performed thrice in triplicate. Bars represent the mean ± standard deviation of three independent experiments. *p < 0.05, **p < 0.01, and ***p < 0.001.

Previous studies have reported that *rseA*, *rpoE*, *rseB*, *degS*, and *clpP* genes were involved in regulating the ability to resist heat shock in *E. coli* and RseA protein inhibited the activity of RpoE (Missiakas et al., 1997; Kim, 2015). In addition, Zhang et al. (2016) confirmed that the *htrA* gene enhanced the ability to resist heat shock in *G. parasuis*. To study the role of the quorum sensing system in regulating these genes, the differential expression of these genes was quantified using RT-qPCR. The RT-qPCR results showed that the expression of *rseA* gene was significantly up-regulated, whereas the expression of *rpoE*, *rseB*, *degS*, *clpP*, and *htrA* genes was down-regulated obviously in ΔluxS, compared with GPS2. The same results were also found between ΔluxS and C-luxS strains. However, no significant differential expression was present between GPS2 and C-luxS strains (Figure 4A). These results suggested that the quorum sensing system could regulate the ability to resist heat shock of *G. parasuis via* regulating the expression of heat shock-related genes. Additionally, *rseA* and *rpoE* genes may exert an opposite effect in regulating the toleration of heat shock resistance in *G. parasuis*.

To confirm that *rseA* and *rseB* genes exerted opposite effects in regulating the toleration of heat shock resistance in *G. parasuis*, *rseA* and *rpoE* mutant strains were constructed. The PCR identification results of GPS2, ΔrseA, and ΔrpoE strains were shown in Figures 4B,C. As expected, the UKD sequence and kanamycin resistance cassette sequence could be amplified, in the *rseA* and *rpoE* mutant strains, whereas the *rseA* and *rpoE* genes could not be amplified. These results indicated that *rseA* and *rpoE* mutant strains were constructed successfully, as confirmed by sequencing.

To better understand the regulating relationship of these heat shock-related genes, the expression of *rseA*, *rpoE*, *rseB*, *degS*, *clpP*, and *htrA* genes was also quantified among GPS2, ΔrseA, and ΔrpoE strains. The results showed that *rseA* gene was down-regulated in the ΔrpoE strain and the *rpoE* gene was up-regulated in the ΔrseA strain, compared with the wild type strain. In addition, the expression of *rseB*, *degS*, *clpP*, and *htrA* genes was up-regulated in ΔrseA and down-regulated in ΔrpoE, compared with the GPS2 strain (Figure 4D). These results indicated that *rseA* and *rpoE* genes exerted an opposite regulatory effect on heat shock resistance. Therefore, we hypothesized that ΔrseA could exhibit well ability to resist heat shock, while ΔrpoE displayed the opposite phenotype, compared with wild type.

### Abilities of GPS2, ΔrseA, and ΔrpoE strains to resist heat shock

**Figure 5:**
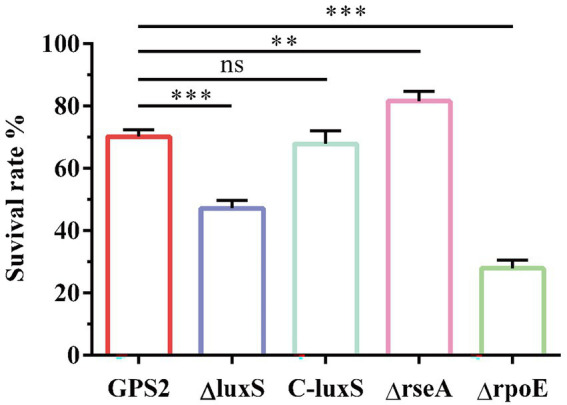
The survival rates of GPS2, ΔluxS, C-luxS, ΔrseA, and ΔrpoE strains under heat shock conditions. All above assays were performed thrice in triplicate. Bars represent the mean ± standard deviation of three independent experiments. **p < 0.01, and ***p < 0.001.

To confirm our hypothesis, the survival rates of wild type strain, ΔluxS, C-luxS, ΔrseA, and ΔrpoE strains under heat shock conditions were measured. The results showed that the survival rates of GPS2, ΔluxS, C-luxS, ΔrseA, and ΔrpoE strains to heat shock stress were 70.2%, 48.1%, 67.9%, 81.7%, and 27.9%, respectively (Figure 5). These results suggested that the deletion of the resA gene enhanced the tolerance of heat shock resistance of *G. parasuis*, whereas the deletion of the rpoE reduced its tolerance to heat shock, compared with the wild type strain. These results further confirmed that quorum sensing system regulates the expression of heat shock-related genes to affect the tolerance of heat stress resistance in *G. parasuis*.

## Discussion

The quorum sensing system is associated with a diverse array of physiological activities and abilities, such as symbiosis, virulence, conjugation, antibiotic production, motility, sporulation, and biofilm formation in *G. parasuis*, *Actinobacillus pleuropneumoniae*, *Glaesserella influenza*, and *Streptococcus mutans* (Miller and Bassler, 2001; Merritt et al., 2003; Daines et al., 2005; Li et al., 2008; Zhang et al., 2019). However, the mechanism by which quorum sensing system regulates the resistance to heat shock remains unclear. Therefore, we analyzed the regulatory networks associated with the LuxS quorum sensing system of *G. parasuis* to reveal the potential regulatory mechanisms. To our knowledge, this is the first study analyzing the function of quorum sensing system in *Glaesserella parasuis* using RNA-seq.

**Figure 6:**
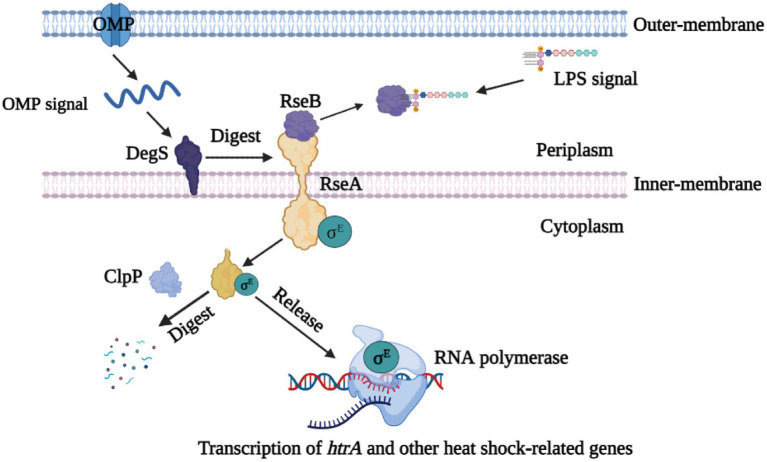
Schematic drawing of the σ^E^ signaling pathway. As stress sensor proteins, DegS protease is activated by the binding of the OMP C-terminus, and RseB is relieved from RseA by the accumulation of LPS in the periplasm. RseA is sequentially digested by DegS and ClpP, thereby releasing σ^E^ in the cytoplasm. Finally, σ^E^ along with RNA polymerase can induce the transcription of the *htrA* gene and other heat shock-relate genes.

Although we have previously shown that the resistance of quorum sensing system deficient *G. parasuis* to heat shock is weaker than that of wild type strain (Zhang et al., 2019), the underlying regulatory mechanism is still unclear. The transcriptome results showed that the *rseA* gene was up-regulated, whereas *rseB*, *degS*, *clpP*, *rpoE*, and *htrA* genes were down-regulated in ΔluxS, compared with the wild type strain. All these genes have been reported to be involved in the regulation of resistance to heat shock in *E. coli* (Kim, 2015). Therefore, based on our transcriptome results, we hypothesized that quorum sensing system regulates the resistance of *G. parasuis* to heat as follows: under normal conditions, DegS protease is activated by the binding of the outer membrane protein (OMP) C-terminus, and RseB protein is relieved from RseA by the accumulation of lipopolysaccharide (LPS) in the periplasm (Mecsas et al., 1993; Sohn et al., 2007). Next, RseA is sequentially digested by DegS and ClpP, thereby releasing σ^E^ to the cytoplasm, which is produced by RpoE (Missiakas et al., 1997; Chaba et al., 2011; Lima et al., 2013). The released σ^E^ in combination with HtrA increases the ability of *G. parasuis* to heat shock (Zhang et al., 2016). Finally, the compound can play a positive regulatory effect in the heat shock resistance mechanism (Figure 6). However, when the *luxS* gene was deleted, the expression of heat shock-related genes is altered, thus suppressing the release of σ^E^ and expression of the *htrA* gene, and finally reducing the ability of *G. parasuis* to resist heat shock. And the regulatory network of quorum sensing system to heat shock resistance in *G. parasuis* requires to in-depth study.

GO categories results showed that amino acid metabolic process and catalytic activity were down-regulated in the ΔluxS strain, which is consistent with the finding of a previous report that implicated quorum sensing in controlling the amino acid metabolism (Withers et al., 2001). The LuxS protease plays an important role in the activated methyl cycle (AMC) which is a pivotal metabolic pathway that recycles homocysteine from S-adenosyl methionine (SAM) to maintain the *de novo* methionine biosynthesis (Hardie and Heurlier, 2008). Therefore, the change in the activated methyl cycle could contribute to the downregulation of the amino acid metabolic process and catalytic activity. KEGG pathway analyzing results showed ABC transporters as one of the most abundant pathways of up-regulated genes in ΔluxS strain. ABC transporters are involved in the secretion of antibiotics through the cell membrane and also contribute to acquired antibiotic resistance (Mendez and Salas, 2001), which explains the finding that the quorum sensing system is associated with antibiotic production (Miller and Bassler, 2001).

## Conclusion

In summary, RNA-seq was conducted to compare the differences in transcriptome and illustrate the regulatory effect of the quorum sensing system in GPS2, ΔluxS, and C-luxS strains. Four hundred forty-four differentially expressed genes were identified in the ΔluxS mutant strain, compared with GPS2. Several of these genes were involved in the molecular signaling pathway interactions with the quorum sensing system. In addition, we demonstrated that *G. parasuis* could utilize the quorum sensing system to regulate its heat shock resistance. We further confirmed that *rseA* and *rpoE* genes exerted an opposite regulating effect in heat shock resistance. In conclusion, we believe that this study provides first-hand information regarding the molecular mechanism by which the quorum sensing system regulates the heat shock resistance of *G. parasuis*.

## Data availability statement

The RNA‐seq data are available in the NCBI Sequence Read Archive (SRA) with accession number PRJNA859659 (https://www.ncbi.nlm.nih.gov/sra/, PRJNA859659).

## Author contributions

BZ, CJ, WL, and QH: conceptualization and writing—review and editing. CJ and JR: data curation. QH: funding acquisition, project administration, and supervision. BZ and CJ: investigation and writing—original draft. BZ, CJ, HC, WZ, and YH: methodology. BZ, CJ, HC, WZ, and JR: visualization. All authors contributed to the article and approved the submitted version.

## Funding

This research was supported by China Agriculture Research System of MOF and MARA (No. CARS-35).

## Conflict of interest

The authors declare that the research was conducted in the absence of any commercial or financial relationships that could be construed as a potential conflict of interest.

## Publisher’s note

All claims expressed in this article are solely those of the authors and do not necessarily represent those of their affiliated organizations, or those of the publisher, the editors and the reviewers. Any product that may be evaluated in this article, or claim that may be made by its manufacturer, is not guaranteed or endorsed by the publisher.

## Footnotes

^1^ https://www.ncbi.nlm.nih.gov/nuccore/CP001321.1

^2^ http://www.geneontology.org

^3^ http://www.genome.jp/kegg/

## References

[ref1] Abisado R. G. Benomar S. Klaus J. R. Dandekar A. A. Chandler J. R. (2018). Bacterial quorum sensing and microbial community interactions. MBio 9, e02331–17. doi: 10.1128/mBio.01749-18 29789364 PMC5964356

[ref2] Chaba R. Alba B. M. Guo M. S. Sohn J. Ahuja N. Sauer R. T.. (2011). Signal integration by DegS and RseB governs the sigma(E)-mediated envelope stress response in *Escherichia coli*. Proc. Natl. Acad. Sci. U. S. A. 108, 2106–2111. doi: 10.1073/pnas.1019277108, PMID: 21245315 PMC3033255

[ref3] Chitlaru T. Zaide G. Ehrlich S. Inbar I. Cohen O. Shafferman A. (2011). HtrA is a major virulence determinant of *Bacillus anthracis*. Mol. Microbiol. 81, 1542–1559. doi: 10.1111/j.1365-2958.2011.07790.x, PMID: 21801240

[ref4] Daines D. A. Bothwell M. Furrer J. Unrath W. Nelson K. Jarisch J.. (2005). *Haemophilus influenzae* luxS mutants form a biofilm and have increased virulence. Microb. Pathog. 39, 87–96. doi: 10.1016/j.micpath.2005.06.003, PMID: 16099134

[ref5] Fu S. L. Guo J. Li R. Z. Qiu Y. S. Ye C. Liu Y.. (2018). Transcriptional profiling of host cell responses to virulent *Haemophilus parasuis*: new insights into pathogenesis. Int. J. Mol. Sci. 19, 19. doi: 10.3390/ijms19051320 PMC5983834 29710817

[ref6] Hardie K. R. Heurlier K. (2008). Establishing bacterial communities by 'word of mouth': LuxS and autoinducer 2 in biofilm development. Nat. Rev. Microbiol. 6, 635–643. doi: 10.1038/nrmicro1916, PMID: 18536728

[ref7] Huang J. C. Wang X. R. Cao Q. Feng F. F. Xu X. J. Cai X. W. (2016). ClpP participates in stress tolerance and negatively regulates biofilm formation in *Haemophilus parasuis*. Vet. Microbiol. 182, 141–149. doi: 10.1016/j.vetmic.2015.11.020, PMID: 26711041

[ref8] Kendall M. M. Sperandio V. (2007). Quorum sensing by enteric pathogens. Curr. Opin. Gastroenterol. 23, 10–15. doi: 10.1097/MOG.0b013e3280118289 17133078

[ref9] Kim D. Y. (2015). Two stress sensor proteins for the expression of sigmaE regulon: DegS and RseB. J. Microbiol. 53, 306–310. doi: 10.1007/s12275-015-5112-6, PMID: 25935301

[ref10] Li L. Zhou R. Li T. T. Kang M. S. Wan Y. Xu Z. F.. (2008). Enhanced biofilm formation and reduced virulence of *Actinobacillus pleuropneumoniae* luxS mutant. Microb. Pathog. 45, 192–200. doi: 10.1016/j.micpath.2008.05.008, PMID: 18585450

[ref11] Lima S. Guo M. S. Chaba R. Gross C. A. Sauer R. T. (2013). Dual molecular signals mediate the bacterial response to outer-membrane stress. Science 340, 837–841. doi: 10.1126/science.1235358, PMID: 23687042 PMC3928677

[ref12] Liu Y. Y. Chen P. Wang Y. Li W. T. Cheng S. Wang C. M.. (2012). Transcriptional profiling of *Haemophilus parasuis* SH0165 response to Tilmicosin. Microb. Drug Resist. 18, 604–615. doi: 10.1089/mdr.2012.0047, PMID: 22935051

[ref13] Liu H. Xue Q. Zeng Q. Zhao Z. (2016). *Haemophilus parasuis* vaccines. Vet. Immunol. Immunopathol. 180, 53–58. doi: 10.1016/j.vetimm.2016.09.002 27692096

[ref14] Ma Y. P. Hao L. Ke H. Liang Z. L. Ma J. Y. Liu Z. X.. (2017). LuxS/AI-2 in *Streptococcus agalactiae* reveals a key role in acid tolerance and virulence. Res. Vet. Sci. 115, 501–507. doi: 10.1016/j.rvsc.2017.07.032, PMID: 28858764

[ref15] Mecsas J. Rouviere P. E. Erickson J. W. Donohue T. J. Gross C. A. (1993). The activity of sigma E, an *Escherichia coli* heat-inducible sigma-factor, is modulated by expression of outer membrane proteins. Genes Dev. 7, 2618–2628. doi: 10.1101/gad.7.12b.2618, PMID: 8276244

[ref16] Mendez C. Salas J. A. (2001). The role of ABC transporters in antibiotic-producing organisms: drug secretion and resistance mechanisms. Res. Microbiol. 152, 341–350. doi: 10.1016/S0923-2508(01)01205-0, PMID: 11421281

[ref17] Merritt J. Qi F. X. Goodman S. D. Anderson M. H. Shi W. Y. (2003). Mutation of luxS affects biofilm formation in *Streptococcus mutans*. Infect. Immun. 71, 1972–1979. doi: 10.1128/IAI.71.4.1972-1979.2003, PMID: 12654815 PMC152054

[ref18] Miller M. B. Bassler B. L. (2001). Quorum sensing in bacteria. Annu. Rev. Microbiol. 55, 165–199. doi: 10.1146/annurev.micro.55.1.165 11544353

[ref19] Missiakas D. Mayer M. P. Lemaire M. Georgopoulos C. Raina S. (1997). Modulation of the *Escherichia coli* sigma(E) (RpoE) heat-shock transcription-factor activity by the RseA, RseB and RseC proteins. Mol. Microbiol. 24, 355–371. 9159522 10.1046/j.1365-2958.1997.3601713.x

[ref20] Mutunga M. Graham S. De Hormaeche R. D. Musson J. A. Robinson J. H. Mastroeni P.. (2004). Attenuated *Salmonella typhimurium* htrA mutants cause fatal infections in mice deficient in NADPH oxidase and destroy NADPH oxidase-deficient macrophage monolayers. Vaccine 22, 4124–4131. doi: 10.1016/j.vaccine.2003.10.053, PMID: 15364466

[ref21] Nealson K. H. Hastings J. W. (1979). Bacterial bioluminescence: its control and ecological significance. Microbiol. Rev. 43, 496–518. doi: 10.1128/mr.43.4.496-518.1979, PMID: 396467 PMC281490

[ref22] Pallen M. J. Wren B. W. (1997). The HtrA family of serine proteases. Mol. Microbiol. 26, 209–221. doi: 10.1046/j.1365-2958.1997.5601928.x 9383148

[ref23] Sohn J. Grant R. A. Sauer R. T. (2007). Allosteric activation of DegS, a stress sensor PDZ protease. Cell 131, 572–583. doi: 10.1016/j.cell.2007.08.044, PMID: 17981123

[ref24] Wang X. R. Xu X. J. Wu Y. C. Li L. Y. Cao R. J. Cai X. W.. (2013). Polysaccharide biosynthesis protein CapD is a novel pathogenicity-associated determinant of *Haemophilus parasuis* involved in serum-resistance ability. Vet. Microbiol. 164, 184–189. doi: 10.1016/j.vetmic.2013.01.037, PMID: 23434184

[ref25] Waters C. M. Bassler B. L. (2005). Quorum sensing: cell-to-cell communication in bacteria. Annu. Rev. Cell Dev. Biol. 21, 319–346. doi: 10.1146/annurev.cellbio.21.012704.131001 16212498

[ref26] Wen Z. T. Nguyen A. H. Bitoun J. P. Abranches J. Baker H. V. Burne R. A. (2011). Transcriptome analysis of LuxS-deficient *Streptococcus mutans* grown in biofilms. Mol. Oral. Microbiol. 26, 2–18. doi: 10.1111/j.2041-1014.2010.00581.x, PMID: 21214869 PMC3105442

[ref27] Withers H. Swift S. Williams P. (2001). Quorum sensing as an integral component of gene regulatory networks in Gram-negative bacteria. Curr. Opin. Microbiol. 4, 186–193. doi: 10.1016/S1369-5274(00)00187-9, PMID: 11282475

[ref28] Xie F. Zhang Y. H. Li G. Zhou L. Liu S. G. Wang C. L. (2013). The ClpP protease is required for the stress tolerance and biofilm formation in *Actinobacillus pleuropneumoniae*. PLoS One 8:e53600. doi: 10.1371/annotation/fbe62755-866e-4a4e-baf8-4bb40019a8fb 23326465 PMC3543445

[ref29] Xue T. Zhao L. P. Sun B. L. (2013). LuxS/AI-2 system is involved in antibiotic susceptibility and autolysis in *Staphylococcus aureus* NCTC 8325. Int. J. Antimicrob. Agents 41, 85–89. doi: 10.1016/j.ijantimicag.2012.08.016, PMID: 23084594

[ref30] Yuan L. H. Hillman J. D. Progulske-Fox A. (2005). Microarray analysis of quorum-sensing-regulated genes in *Porphyromonas gingivalis*. Infect. Immun. 73, 4146–4154. doi: 10.1128/IAI.73.7.4146-4154.2005, PMID: 15972504 PMC1168601

[ref31] Zhang B. He Y. B. Xu C. G. Xu L. N. Feng S. X. Liao M.. (2012). Cytolethal distending toxin (CDT) of the *Haemophilus parasuis* SC096 strain contributes to serum resistance and adherence to and invasion of PK-15 and PUVEC cells. Vet. Microbiol. 157, 237–242. doi: 10.1016/j.vetmic.2011.12.002, PMID: 22221379

[ref32] Zhang B. Ku X. Zhang X. Zhang Y. Chen G. Chen F.. (2019). The AI-2/luxS quorum sensing system affects the growth characteristics, biofilm formation, and virulence of *Haemophilus parasuis*. Front. Cell. Infect. Microbiol. 9:62. doi: 10.3389/fcimb.2019.00062, PMID: 30941317 PMC6434701

[ref33] Zhang L. H. Li Y. Wen Y. P. Lau G. W. Huang X. B. Wu R.. (2016). HtrA is important for stress resistance and virulence in *Haemophilus parasuis*. Infect. Immun. 84, 2209–2219. doi: 10.1128/IAI.00147-16, PMID: 27217419 PMC4962635

[ref34] Zou Y. Feng S. X. Xu C. G. Zhang B. Zhou S. M. Zhang L. Y.. (2013). The role of galU and galE of *Haemophilus parasuis* SC096 in serum resistance and biofilm formation. Vet. Microbiol. 162, 278–284. doi: 10.1016/j.vetmic.2012.08.006, PMID: 22981816

